# Gap junction remodeling associated with cholesterol redistribution during fiber cell maturation in the adult chicken lens

**Published:** 2009-08-04

**Authors:** Sondip K. Biswas, Jean X. Jiang, Woo-Kuen Lo

**Affiliations:** 1 Department of Neurobiology, Morehouse School of Medicine, Atlanta, GA; 2 Department of Ophthalmology, Emory University, Atlanta, GA; 3 Department of Biochemistry, University of Texas Health Science Center at San Antonio, TX

## Abstract

**Purpose:**

To investigate the structural remodeling in gap junctions associated with cholesterol redistribution during fiber cell maturation in the adult chicken lens. We also evaluated the hypothesis that the cleavage of the COOH-terminus of chick Cx50 (formerly Cx45.6) during fiber cell maturation would affect the gap junction remodeling.

**Methods:**

Freeze-fracture TEM and filipin cytochemistry were applied to visualize structural remodeling of gap junction connexons associated with cholesterol redistribution during fiber cell maturation in adult leghorn chickens (42-62 weeks old). Freeze-fracture immunogold labeling (FRIL) was used to label the specific Cx50 COOH-terminus antibody in various structural configurations of gap junctions.

**Results:**

Cortical fiber cells of the adult lenses contained three subtypes of cholesterol-containing gap junctions (i.e., cholesterol-rich, cholesterol-intermediate, and cholesterol-poor or -free) in both outer and inner cortical zones. Quantitative studies showed that approximately 81% of gap junctions in the outer cortex were cholesterol-rich gap junctions whereas approximately 78% of gap junctions in the inner cortex were cholesterol-free ones. Interestingly, all cholesterol-rich gap junctions in the outer cortex displayed loosely-packed connexons whereas cholesterol-free gap junctions in the deep zone exhibited tightly, hexagonal crystalline-arranged connexons. Also, while the percentage of membrane area specialized as gap junctions in the outer cortex was measured approximately 5 times higher than that of the inner cortex, the connexon density of the crystalline-packed gap junctions in the inner cortex was about 2 times higher than that of the loosely-packed ones in the outer cortex. Furthermore, FRIL demonstrated that while the Cx50 COOH-terminus antibody was labeled in all loosely-packed gap junctions examined in the outer cortex, little to no immunogold labeling was seen in the crystalline-packed connexons in the inner cortex.

**Conclusions:**

Gap junctions undergo significant structural remodeling during fiber cell maturation in the adult chicken lens. The cholesterol-rich gap junctions with loosely-packed connexons in the young outer cortical fibers are transformed into cholesterol-free ones with crystalline-packed connexons in the mature inner fibers. In addition, the loss of the COOH-terminus of Cx50 seems to contribute equally to the transformation of the loosely-packed connexons to the crystalline-packed connexons during fiber cell maturation. This transformation causes a significant increase in the connexon density in crystalline gap junctions. As a result, it compensates considerably for the large decrease in the percentage of membrane area specialized as gap junctions in the mature inner fibers in the adult chicken lens.

## Introduction

The lens fiber cell membrane contains the richest cholesterol content in the body [1-6]. The cholesterol to phospholipids (C/P) molar ratio ranges from 1 to 4 from the lens cortex to lens nucleus, while that of typical eukaryotic cells is between 0.5 and 1.0 [1]. Our previous study has shown that the newly-formed gap junctions are enriched with cholesterol in the cortical fiber cells because they are assembled in the cholesterol-rich fiber cell membrane during lens differentiation [7]. Since the gap junctions of lens fibers do not turn over as rapidly as those in other tissues [8-13], the newly formed cholesterol-rich gap junctions would undergo a maturation process during fiber cell differentiation and maturation. Indeed, our early study demonstrated that there is a redistribution of cholesterol in gap junctions during fiber cell differentiation and maturation in the embryonic chicken lens [7]. Specifically, a majority of cholesterol-rich gap junctions in the young outer cortical fibers have transformed into cholesterol-free ones in the mature inner cortical fibers [7].

Formation of gap junctions in young differentiating fiber cells and other cell types has been visualized with freeze-fracture TEM [7,14-18]. Early stage gap junction formation is identified as a cluster of several 8-9 nm intramembrane particles on the P-face of the plasma membrane on freeze-fracture electron micrographs. Each particle represents a single connexon, also known as a hemichannel of gap junctions. Two individual connexons (hemichannels) from two adjacent cells are aligned together in a specific head-to-head manner across the intercellular space to form a complete intercellular gap junction channel. Each connexon is composed of gap junction proteins, called connexins, and six connexin molecules are oligomerized to form one connexon. Oligomerization of connexins into hexameric connexons occurs after the connexins exit the endoplasmic reticulum [19-21]. These connexons are trafficked via the Golgi apparatus to the plasma membrane to form gap junction channels. A continued clustering of gap junction channels eventually generates a large gap junction plaque.

Approximately 20 members of the connexin family have been identified in the human or mouse genome, and multiple connexin isoforms are expressed in most cell types [22,23]. Two connexins have been identified in the lens fibers of various species such as the mouse (Cx46 and Cx50) [24,25], sheep (Cx49 and Cx44) [26-28], and chicken (Cx45.6 and Cx56) [29,30]. In this study, the chick Cx50 (formerly Cx45.6) and Cx46 (formerly Cx56) are used to follow the nomenclature of human and rodent homologues to avoid confusion. Among the several functional domains of the connexin molecule, the COOH-terminal tail domain is highly variable among different connexins and is a site for protein binding and phosphorylation by kinases that directly regulate channel gating [31-34]. Interestingly, in the lens system, the loss of the COOH-terminus of Cx50 has been suggested to be responsible for its pH sensitivity change, and may be crucial to keeping the gap junction channel open for mature fiber cell survival in adult lenses [32,35-37].

The reason for cholesterol redistribution in fiber gap junctions during lens differentiation is not clear. It has been suggested that the presence of cholesterol may be involved in gap junction assembly in other cell types [38-40]. Since the cholesterol molecule inserts itself in the membrane in the same polar head orientation as those of the phospholipids, cholesterol molecules can immobilize the first few hydrocarbon groups of the phospholipid molecules and make the lipid bilayer less deformable. Also, cholesterol can prevent crystallization of hydrocarbons and phase shifts in the membrane, and is therefore an important structural lipid component for controlling the fluidity of the cell membrane [6,41]. It is conceivable that the presence of different amounts of cholesterol in fiber gap junctions would most likely affect the arrangement of their connexons. However, in our early study we have not observed significant changes in the connexon arrangements during fiber cell differentiation in the embryonic chicken lens [7]. Both cholesterol-rich and cholesterol-free gap junctions mainly exhibit the loosely-packed configuration of connexons. Although some cholesterol-free gap junctions display a tighter packing of connexons, they do not show the distinct hexagonal crystalline arrangement [7].

In this study, we used the adult chicken lens to further examine the possible structural changes of gap junctions in relation to their cholesterol redistribution during fiber cell maturation and aging. We conducted systematic structural and quantitative analyses on the distribution of cholesterol in gap junctions from superficial to deep cortical fiber cells by filipin cytochemistry. In addition, freeze-fracture immunogold labeling (FRIL) was used to evaluate the hypothesis that the loss of the COOH-terminus of chick Cx50 (formerly Cx45.6) may affect the structural remodeling of gap junctions during fiber cell maturation. We have found that gap junctions indeed undergo significant structural remodeling during fiber cell maturation and aging in the adult lens. In the superficial young fibers, the gap junctions are enriched with cholesterol, and their connexons are loosely-packed. In the deep mature fibers, the gap junctions are devoid of cholesterol, and their connexons are tightly crystalline-packed. In addition, we have revealed that while the percentage of membrane area specialized as gap junctions is significantly decreased as fiber cells mature, the connexon density of the crystalline-packed gap junctions in the mature inner fibers is approximately two times higher than that of the loosely-packed ones in the young outer fibers. Moreover, FRIL analysis has shown that the COOH-terminus of Cx50 is lost in most gap junctions with crystalline-packed connexons in the mature fiber cells. This study suggests that redistribution of cholesterol, together with the loss of the Cx50 COOH-terminus, may regulate the specific packing and density of connexons in gap junctions during fiber cell maturation in the adult chicken lens.

## Methods

### Collection of adult chicken lenses

Adult white leghorn chickens (42-62 weeks old) were purchased from a local poultry farm (Hyline International, Mansfield, GA). All lenses were immediately removed from freshly isolated eyes and processed for the various experiments described below. The animals were treated in accordance with the Association for Research in Vision and Ophthalmology Resolution on the Use of Animals in Research.

### Freeze-fracture TEM and cytochemical detection of cholesterol

Freshly isolated lenses were fixed in 2.5% glutaraldehyde in 0.1M cacodylate buffer (pH 7.3) at room temperature (RT) for 2-4 h. After washing in buffer, lenses were orientated to obtain sagittal (longitudinal) sections using a vibratome, and slices were collected, marked serially from superficial to deep, and kept separately. Because the dimension of each vibratome slice was too large for a routine freeze fracturing, the areas of interest were carefully dissected into small rectangular or square blocks (~2x2 mm) from the anterior-central or posterior-central surface of lens slices. These slices were then cryoprotected with 25% glycerol in 0.1 M cacodylate buffer at RT for 1 h and processed for freeze-fracture TEM according to our routine procedures [7]. In brief, a single lens slice was mounted on a gold specimen carrier and frozen rapidly in liquefied Freon 22 and stored in liquid nitrogen. Cryofractures of frozen slices were made in a modified Balzers 400T freeze-fracture unit (Boeckeler Instruments, Inc., Tucson, Arizona), at a stage temperature of -135 ^o^C in a vacuum of approximately 2x10^^-7^^ Torr. The lens tissue was fractured by scraping a steel knife across a frozen surface to explore fiber cell membranes. The fractured surface was immediately replicated with platinum (~2 nm thick) followed by carbon film (~25 nm thick). The replicas, obtained by unidirectional shadowing, were cleaned with household bleach and examined with a transmission electron microscope.

For cytochemical detection of filipin-cholesterol-complexes (FCCs) with freeze-fracture TEM, we followed the previous procedures [42,43] with modifications [7]. In brief, the vibratome slices were incubated in a mixture of 2.5% glutaraldehyde in 0.1 M cacodylate buffer and 0.1% filipin in dimethyl formamide (Sigma, St. Louis, MO) for 24 h. The slices were then cryoprotected with 25% glycerol in 0.1 M cacodylate buffer at RT for 1 h and processed for conventional freeze-fracture TEM described above. The FCCs are discrete particles or pits (25-35 nm in diameter) which can be clearly visualized on the P face and E face of the plasma membrane with freeze-fracture TEM. The formation of FCCs is due to the polyene antibiotic filipin reacting specifically with membrane cholesterol which produces characteristic membrane lesions seen as the FCCs. In order to avoid possible artifacts due to insufficient incubation time for filipin to diffuse into the entire lens slices to react with membrane cholesterol, we have tested a number of incubation times, ranging from 8-48 h and obtained similar results for each time point tested. We thus used a 24 h incubation time, convenient to our experiments. In addition, based on our previous experience [7], we have used freshly prepared filipin solution in each experiment to ensure that we achieve the best quality control for consistent results.

### High pressure freezing for freeze-fracture TEM

Freshly isolated lenses from adult chickens were briefly rinsed in Medium 199 at RT and then dissected with a pair of sharp scissors into small rectangular blocks (approximately 6×2 mm) from superficial to deep cortical regions of the lens. Two gold specimen carriers (Leica, Inc., Bannockburn, IL), each with a cylinder-shaped indentation, were used together to sandwich a lens block in between the carriers for high-pressure freezing. In order to make the lens block stick to only one carrier during freezing, one of the carriers was treated with 10% lecithin in chloroform for 15 min and dried for 30 min before use. Also, in order to prevent any air bubbles from forming at the surface of the lens block, the specimen was treated with 100% 1-hexadecene (Sigma) before being mounted in the carriers. The lens block inside the specimen carriers was carefully transferred into the sample holder and immediately frozen with a Balzers HPM010 high pressure freezing machine (Boeckeler Instruments, Inc.). All high-pressure freezing experiments were conducted at the Integrated Microscopy and Microanalytical Facility at Emory University, Atlanta, GA. The frozen specimens were stored in liquid nitrogen before processing for freeze-fracture TEM. In our separate experiments, freshly isolated lenses from the adult rats and mice were also processed by the same manner for high pressure freezing and freeze-fracture TEM.

### Freeze-fracture immunogold labeling (FRIL)

Freshly isolated chicken lenses were lightly fixed in 0.75% paraformaldehyde in PBS for 30-45 min at RT, then cut into 300 μm slices with a Vibratome to make freeze-fracture replicas. One drop of 0.5% parloidion in amyl acetate was used to secure the integrity of the whole piece of a large replica during cleaning and immunogold labeling procedures. The replica was washed with 2.5% sodium dodecyl sulfate, 10 mM Tris-HCl, 30mM sucrose, pH 8.3 (SDS buffer) at 50 ^o^C until all visible attached tissue debris was removed from the replica. The replica was then rinsed with PBS, blocked with 4% BSA-0.5% teleostean gelatin in PBS for 30 min and incubated with affinity purified rabbit anti-Cx50 polyclonal antibody made from the COOH-terminus (amino acids 237-400) [36] at 1:10 dilution for 1 h at RT. The replica was washed with PBS and incubated with 10 nm Protein A gold (EY Laboratories, San Mateo, CA) at 1:50 dilution for 1 h at RT. After rinsing, the replica was fixed in 0.5% glutaraldehyde in PBS for 10 min, rinsed in water, collected on a 200 mesh Gilder finder grid, rinsed with 100% amyl acetate for 30 s to remove parloidion and viewed with a JEOL 1200EX TEM.

### Quantitative analyses

#### Changes in the size and percentage of membrane area specialized as gap junctions from the outer to inner cortical fibers

**Figure 1:**
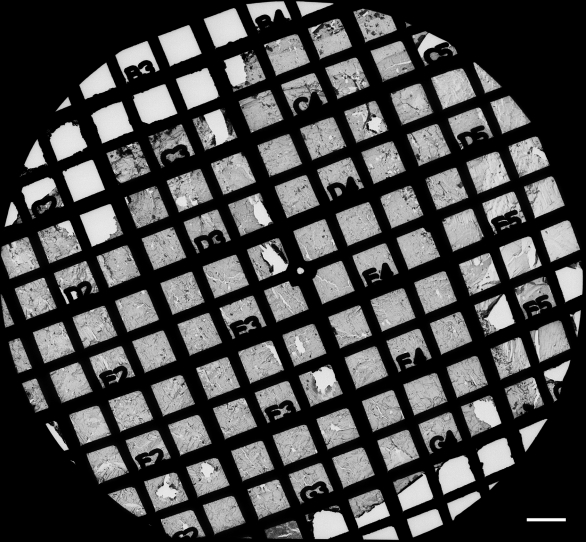
Overview of an intact freeze-fracture replica on the Girder finder grid with index number for systematic examinations of fiber gap junctions in the adult chicken lens. This representative large intact replica was prepared from a mid-sagittal Vibratome lens section (300 μm thick), which was initially trimmed into approximately 2×2 mm blocks to include the superficial and deep cortices from the anterior central region of the lens. Initial identifications of the lens regions (i.e., superficial versus deep) were made and recorded on the intact replica (~1,000 μm in diameter) using the index number. Detailed examinations of the structures of interest were focused primarily on the outer cortex (0-400 μm) and the inner cortex (400-800 μm from the surface) of the lens replica. The scale bar indicates 100 μm.

Since gap junctions are continuously formed during fiber cell differentiation and maturation, we were interested in seeing whether the size and percentage of membrane area specialized as gap junctions undergo any significant changes during these processes. The micrographs of gap junctions at 30,000X were taken randomly from flat cell membranes in the outer cortex and inner cortex from three large freeze-fracture replicas (Figure 1). The size of gap junctions was measured with the Zeiss AxioVision LE 4.4 on PC (Zeiss Inc., Thornwood, NY). The percentage of cell membrane area specialized as gap junctions was calculated from micrographs taken from three individual replicas. Statistical comparisons were made by T-test using the software SPSS 14.0 (SPSS Inc., Chicago, IL). A p <0.05 was considered significant.

#### Changes in the connexon density from the loosely-packed to the crystalline-packed gap junctions

To compare the connexon density between the loosely-packed gap junctions and the crystalline-packed ones, the micrographs of gap junctions were taken randomly from flat cell membranes in the outer cortex and inner cortex from three freeze-fracture replicas. The number of connexons was counted manually with a cell counter from the micrographs taken at 30,000X. Each gap junction area (µm^2^) was measured with the Zeiss AxioVision LE 4.4 on PC (Zeiss Inc.). The number of connexons per µm^2^ gap junction (GJ) area was calculated to determine the difference in the connexon density between the loosely-packed and crystalline-packed gap junctions. Statistical comparisons were made by T-test using the software SPSS 14.0 (SPSS Inc.). A p <0.05 was considered significant.

#### Changes in the amounts of filipin-cholesterol complexes (FCCs) in gap junctions from the outer to inner cortical fibers

To determine the changes in the amounts of cholesterol in gap junctions, intact large freeze-fracture replicas were made using mid-sagittal Vibratome sections (300 μm thick slices) prepared from the adult chicken lenses. The Vibratome sections were trimmed into 2×2 mm blocks to include the outer and inner cortices from the central anterior or central posterior region of the lens. The intact whole replicas (~1,000 μm in diameter) were prepared to include the outer cortex (0-400 μm from the surface) and the inner cortex (400-800 μm from the surface). Special Gilder finder grids with index numbers (EM Sciences, Hatfield, PA) were used to identify the cortical regions for systematic examinations. The micrographs of gap junctions at 30,000X were taken randomly from flat cell membranes in the outer and inner cortices from three replicas prepared from three different animals of similar ages. Each gap junction area (µm^2^) was measured with the Zeiss AxioVision LE 4.4 on PC, and the number of FCCs in gap junctions was counted manually with a cell counter. The number of FCCs per µm^2^ GJ area was calculated to determine the subtype of each cholesterol-gap junction measured (i.e., 101-300 and above –cholesterol-rich; 51-100 –cholesterol-intermediate; 0-50 –cholesterol-poor and -free). Statistical comparisons of the mean percentage of cholesterol-containing gap junctions between cortices were made for each subtype by T-test using the software SPSS 14.0 (SPSS Inc.). A p<0.05 was considered significant.

## Results

### Gap junction connexon arrangements undergo significant remodeling during fiber cell maturation

By taking advantage of our advanced freeze-fracture technique, we were able to make many large intact replicas routinely from adult chicken lenses (Figure 1). This allowed us to make systematic examinations on the structural remodeling of gap junction connexons from the outer cortex (0-400 μm from the surface) to the inner cortex (400-800 μm from the surface) in both control and filipin-treated lens fiber tissues (Figure 1).

**Figure 2:**
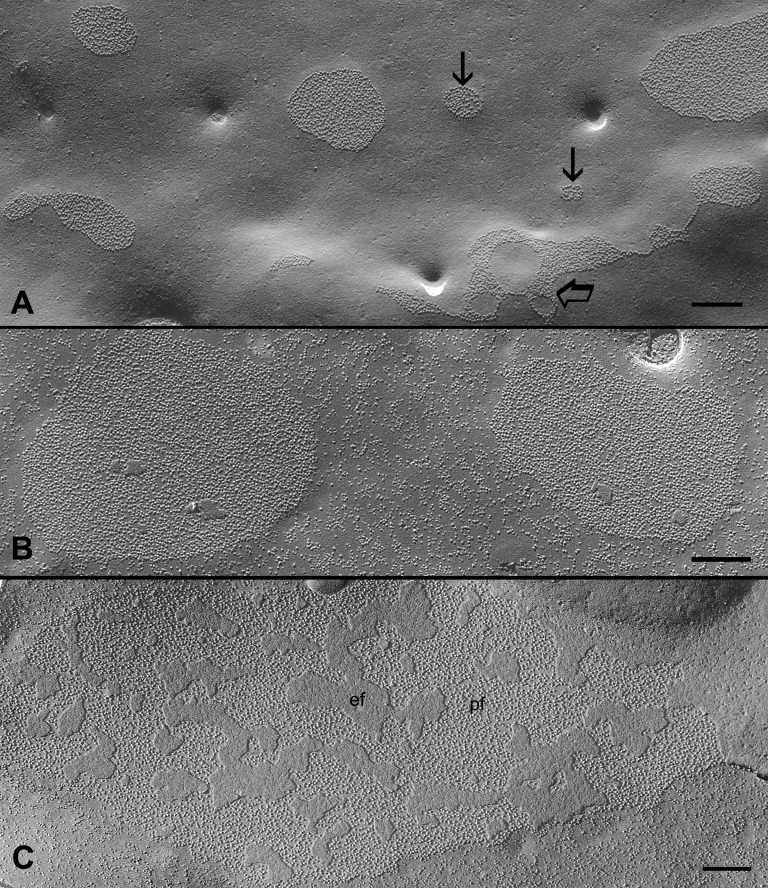
Gap junctions in the outer young cortical fibers (0-400 μm from the surface). **A**: Both small (arrows) and newly-formed gap junctions (opened arrows) in very superficial cells display loosely-packed connexons. **B** & **C**: Several larger gap junction plaques in slightly deeper cells also exhibit loosely-packed connexons. In the images, pf is the P face of the membrane and ef is the E face of the membrane. The scale bars indicate 200 nm.

In the outer cortex, gap junctions were easily found in the differentiating young fiber cells of adult chicken lenses (Figure 2). Some small size gap junctions were particularly irregular in shape and configuration, indicating characteristics of newly-assembled gap junctions (Figure 2A, arrow). As fiber cells mature, many larger size gap junctions were found more frequently in the slightly deeper regions of the outer cortex (Figure 2B,C). It is important to note here that regardless of the size and shape, almost all gap junctions in the outer cortical regions displayed primarily the loosely-packed configuration of connexons (Figure 2A-C).

**Figure 3:**
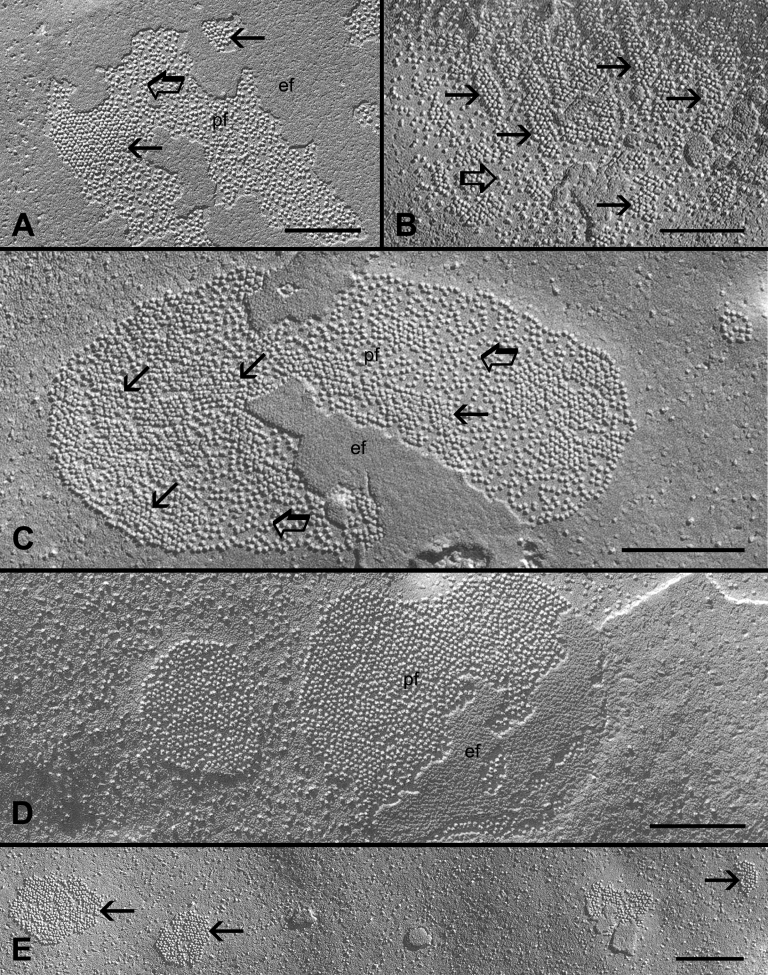
Gap junctions in the inner mature cortical fibers (400-800 μm from the surface) showing several transitional stages of the structural remodeling of gap junctions. **A**: In the more superficial regions, small clusters of crystalline-packed connexons (arrows) are seen mixed with other loosely-packed connexons (opened arrows). **B**: A number of crystalline-packed connexons (arrows) are often aligned into parallel rows within the otherwise loosely-packed (opened arrows) gap junction. **C**: Another example of a gap junction containing a mixture of crystalline-arranged (arrows) and loosely-packed (opened arrows) connexons in which the crystalline-packed configuration is the major form. In the deeper cortical regions, most connexons are primarily in the tightly- or crystalline-packed arrangements (arrows) in both large (**D**) and small (**E**) gap junctions. In the images, pf indicates the P face of membrane and ef indicates the E face of the membrane. The scale bars indicate 200 nm.

The early changes in the connexon packing were seen in the slightly deeper regions of the outer cortex (Figure 3A-C). The connexons of these gap junctions often displayed a mixture of loosely- and crystalline-packed configurations (Figure 3A-C). The crystalline-packed connexons were frequently arranged into small clusters (Figure 3B-C). Finally, the gap junctions with complete tightly- or crystalline-packed connexons were regularly observed in the deeper regions of the inner cortex (Figure 3D,E). Unlike the large gap junctions often seen in the outer cortex (Figure 2C), a number of smaller crystalline gap junctions were frequently found in the inner cortex (Figure 3E).

### Gap junction structural remodeling viewed by high-pressure freezing technique

**Figure 4:**
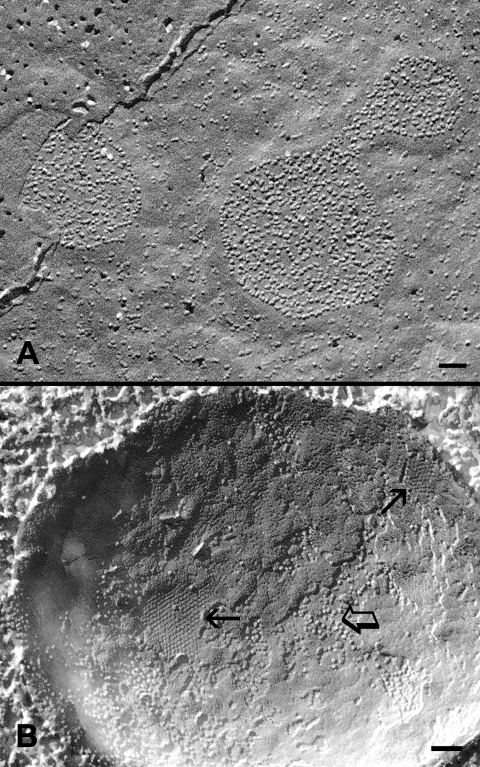
High pressure freezing of gap junctions in cortical fiber cells. Freeze-fracture TEM in conjunction with high pressure freezing shows that while gap junctions display only loosely-packed connexons in superficial cortical fibers (**A**), a mixture of crystalline-packed (arrows) and loosely-arranged (opened arrow) connexons can be visualized in the deeper cortical fibers of the lens without prior chemical fixation (**B**). This additional experimental approach is to confirm that the structural remodeling of gap junctions as observed in the chemically fixed lenses in Figure 2 and Figure 3 is a real change during fiber cell maturation. The scale bars indicate 100 nm.

In order to rule out the possibility that the change in the connexon packing was induced by chemical fixation during tissue preparation [44,45], we applied the high pressure freezing procedures without chemical fixation for our freeze-fracture experiments (see Methods). As predicted, we observed the same pattern of changes in the connexon packing in the adult chicken lens using the high-pressure freezing technique. Specifically, gap junction plaques with the loosely-packed and crystalline-packed connexons were regularly seen in the outer cortex and inner cortex, respectively, in the same replica of the adult chicken lens (Figure 4A,B). Furthermore, in our separate experiments, we have also observed the same crystalline-packed connexons of gap junctions in the deeper cortical fibers of the adult rat and mouse lenses (data not shown).

### Changes in the size and percentage of membrane area specialized as gap junctions during fiber cell maturation

**Figure 5:**
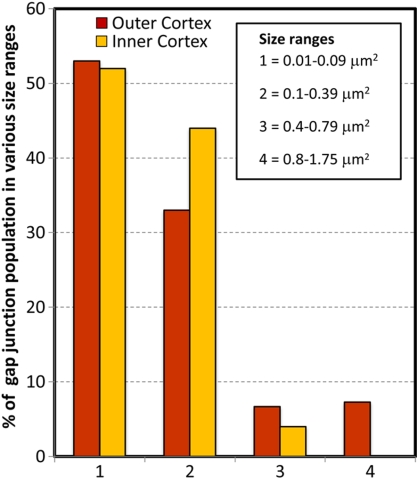
Quantitative analysis of percentage of gap junction population in various size ranges in the outer cortex versus the inner cortex. While the small-size GJs (0.01-0.09 μm^2^) are mostly populated in both outer and inner cortices, the large-size GJs (0.8-1.75 μm^2^) are only found in the outer cortical fibers. Importantly, while all small-size GJs in the outer cortex exhibit loosely-packed connexons (Figure 2), the similar small-size GJs in the inner cortex display the crystalline-packed connexons (Figure 3D). This result suggests that (1) small-size newly-formed GJs are gradually developed into large-size ones during fiber cell differentiation in the outer cortex; and (2) large-size GJs undergo structural remodeling (such as removal of cholesterol and re-packing or breakdown of connexons), which results in a significant reduction in the GJ size during maturation and aging in the inner cortex.

**Figure 6:**
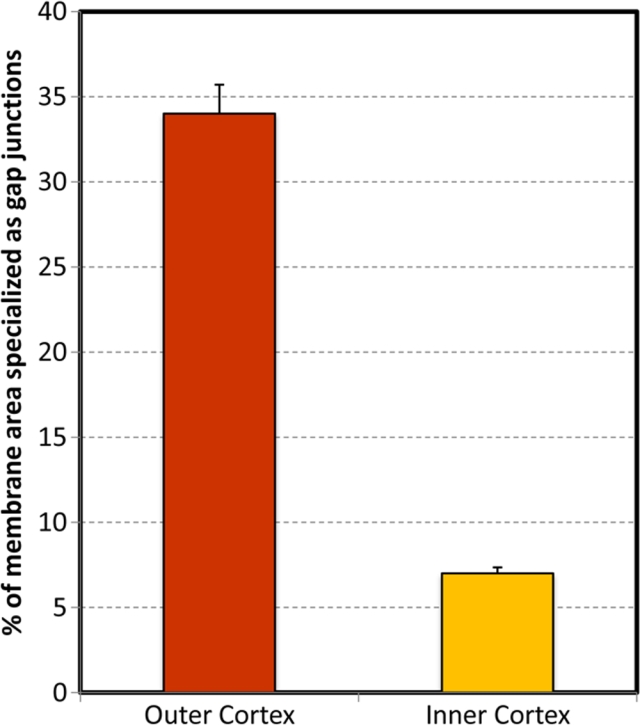
Measurements of the percentage of cell membrane area specialized as gap junctions from the outer cortex to the inner cortex. Quantitative measurements of the percentage of cell membrane area specialized as gap junctions from the outer cortex (0-400 μm from surface) to inner cortex (400-800 μm from surface). The area of gap junctions is significantly reduced by approximately 5 times from the outer cortex (~33%) to inner cortex (~6%). A total of 539 junctions were counted in three replicas in the outer cortex, and 141 gap junctions in three replicas in the inner cortex.

Changes in the size and percentage of membrane area specialized as gap junctions were measured with respect to the change of connexon packing from superficial to deep cortices of the lens. We found that the size and percentage of membrane area specialized as gap junctions decreased during the fiber cell maturation (Figures 5 and Figure 6). Specifically, while many small to medium-size gap junctions could be seen in both outer and inner cortices, the unique large-size group of gap junctions (i.e., 0.8-1.75 µm^2^) was found only in the outer cortex (Figure 2C and Figure 5). As a result, the percentage of membrane area specialized as gap junctions in the outer cortex (33.7%) was significantly higher than that of the inner cortex (6.5%; Figure 6). Since gap junctions in the outer cortex contain typically the loosely-packed connexons whereas those in the inner cortex have mostly the crystalline-packed ones (Figure 2 and Figure 3), we hypothesized that the connexon density of these gap junctions would change during fiber cell maturation.

### Changes in the connexon density of gap junctions during fiber cell maturation

**Figure 7:**
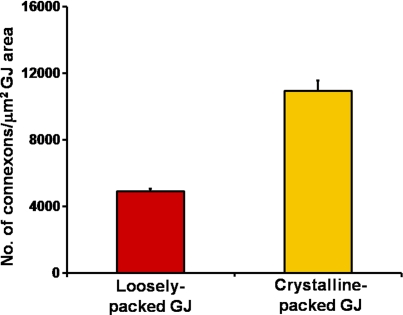
Quantitative comparison of the connexon density between the loosely-packed gap junctions in the outer cortex and the crystalline-packed ones in the inner cortex. The number of connexons in the typical crystalline-packed GJs is approximately 2 times higher than that of the loosely-packed GJs. The connexons from fifteen representative gap junctions of each type from three replicas were counted. This result suggests that although the percentage of membrane area specialized as gap junctions is reduced approximately 5 times from the outer cortex to inner cortex (Figure 6), the number of connexons is reduced only about 2.5 times in the deeper cortical region during fiber cell maturation.

Figure 7 shows that the connexon density (i.e., number of connexons per μm^2^ of gap junction area) of the representative crystalline gap junctions in the inner cortex is approximately two times higher than that of the loosely-packed gap junctions in the outer cortex. This result suggested that the significant increase in the connexon density of the crystalline gap junctions in the inner cortex would compensate considerably for the large decrease in the percentage of membrane area specialized as gap junctions in the inner cortex during fiber cell maturation.

### Different cholesterol distributions in relation to changes in the connexon packing during fiber cell maturation

**Figure 8:**
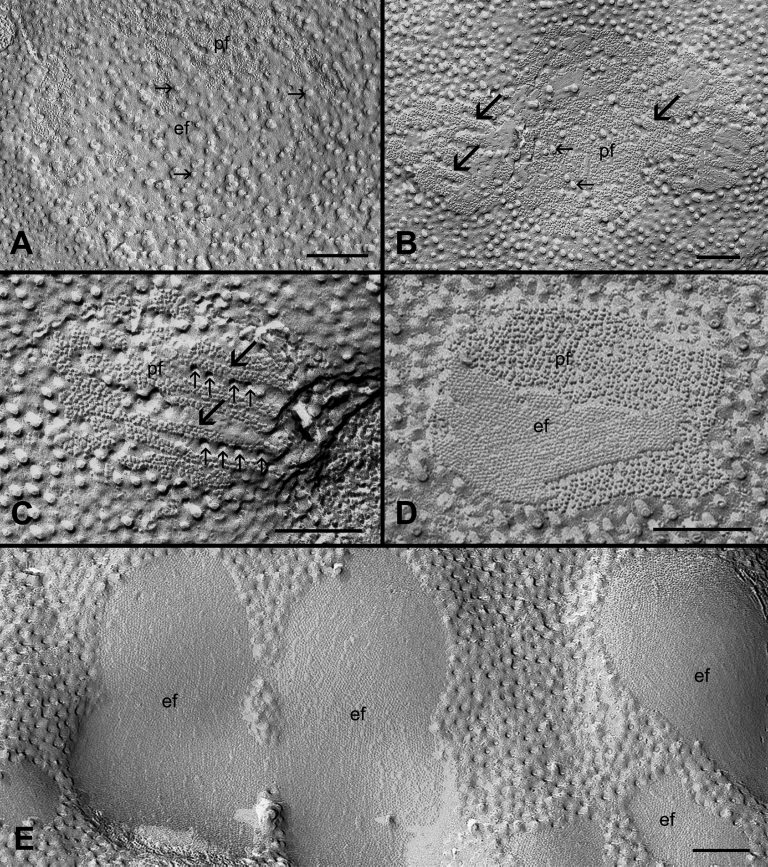
Filipin-treated gap junctions. **A**: A representative cholesterol-rich gap junction with the presence of numerous filipin-cholesterol complexes (FCCs, small arrows) is frequently found in the outer cortical fibers. **B** and **C**: Cholesterol-intermediate gap junctions with less number of FCCs (small arrows) are observed in the inner cortical fibers. FCCs (small arrows) are often distributed along patches or parallel rows of crystalline-packed connexons (large arrows). **D** and **E**: Cholesterol-free gap junctions are mostly seen in the deeper mature cortical fibers. Note that crystalline-arranged connexons can be visualized more clearly on the E-face (ef) of the membrane. In the images, pf indicates the P-face of the membrane. The scale bars indicate 200 nm.

Figure 8 shows the different distributions of cholesterol (i.e., filipin cholesterol complexes, FCCs) in the gap junctions with different packing configurations of connexons seen in the outer and inner cortices of the adult chicken lens. The FCCs are discrete particles or pits (25-35 nm in diameter) that can be clearly visualized on the P face and E face of the plasma membranes with freeze-fracture TEM. The formation of FCC is due to the polyene antibiotic filipin reacting specifically with membrane cholesterol, which produces characteristic membrane lesions seen as the FCC. The cholesterol-rich gap junctions in the outer cortex displayed mainly the loosely-packed connexons (Figure 8A), whereas cholesterol-free gap junctions in the inner cortex exhibit primarily the crystalline-packed connexons (Figure D-E). During this transition, gap junctions with a mixture of loosely- and crystalline-packed connexons were seen to contain rows of FCC particles in between patches of crystalline-packed connexons in the superficial layers of the inner cortex (Figure 8B,C).

### Changes in the amounts of cholesterol in gap junctions during fiber cell maturation

The cholesterol-containing gap junctions were classified into three subtypes, namely cholesterol-rich (101-300 FCCs/µm^2^ GJ area), cholesterol-intermediate (51-100 FCCs/µm^2^ GJ area), and cholesterol-poor-free (0-50 FCCs/µm^2^ GJ area), according to the number of FCCs per μm^2^ of gap junction area. We found that most cholesterol-rich gap junctions were distributed in the outer cortical fiber cells, whereas cholesterol-poor or -free gap junctions were found in the inner cortical fiber cells.

Table 1 shows the quantitative data obtained from three replicas made from three different adult chickens. The data are expressed as mean±SD. In each replica, the percentage of a given cholesterol-GJ subtype is determined by the total GJ area of that given subtype divided by the sum of GJ area from the three subtypes. Quantitative analysis shows that the percentage of cholesterol-rich gap junctions was decreased from the outer cortex (81.2%) to the inner cortex (9.8%), whereas the percentage of cholesterol-poor and -free gap junctions was increased from the outer cortex (4.2%) to the inner cortex (78.1%). A T-test showed that these changes were statistically significant (p<0.001). A total of 539 and 141 gap junctions were analyzed in the outer cortex and inner cortex, respectively. It is worthy of note that although the total membrane area analyzed in the outer cortex versus the inner cortex was comparable, the number of gap junctions found in these two cortical regions was significantly different (see Figure 6). It should also be noted that the main objective of this quantitative analysis was to determine the distribution difference for the three subtypes of cholesterol-gap junctions in these two cortical regions. The absolute number of cholesterol molecules cannot be pursued by this approach and was not within the scope of this study.

**Table 1:** Distribution of the three subtypes of cholesterol-containing gap junctions in the outer and inner cortical fibers of adult chicken lenses.

**Lens area**	**Chole-GJ subtypes (FCC/μm^2^GJ)**	**Mean GJ area (μm^2^)**	**Mean FCC per μm^2^ GJ area**	**Mean percentage of Chole-GJ subtypes**
Outer cortex (0-400 μm from surface)	Chole-poor-free (0-50)	1.31±0.49	32±13	4.2±1.8*
	Chole-intermed. (51-100)	4.41±1.85	80±7	14.5±8.0
	Chole-rich (101-300)	27.25±10.05	210±40	81.2±9.7**
Inner cortex (400-800 μm from surface)	Chole-poor-free (0-50)	4.27±0.26	33±9	78.1±2.3 *
	Chole-intermed. (51-100)	0.66±0.12	77±7	12.0±1.9
	Chole-rich (101-300)	0.54±0.10	188±48	9.8±1.2**

The data are expressed as mean±SD. The percentage of a given cholesterol-GJ subtype in each replica (N=3) is determined by the total GJ area of that given subtype divided by the sum of GJ area from the three subtypes. The asterisk denotes that the increase on the mean percentage of cholesterol-poor-free gap junctions from the outer cortex to inner cortex is statistically significant (p<0.001). The double asterisk denotes that by T-test the decrease on the mean percentage of cholesterol-rich gap junctions from the outer cortex to inner cortex is also statistically significant (p<0.001). A total of 539 and 141 gap junctions were used for quantitative analysis in the outer cortex and inner cortex, respectively. In the table, FCC indicates filipin cholesterol complexes.

### Loss of the COOH-terminus of Cx50 (formerly Cx45.6) in the gap junctions with crystalline-packed connexons during fiber cell maturation

**Figure 9:**
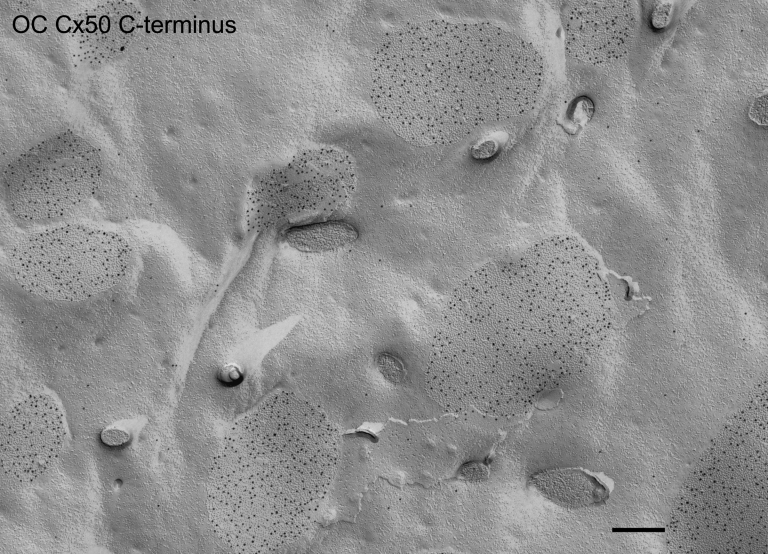
Freeze-fracture replica immunogold labeling. Freeze-fracture replica immunogold labeling (FRIL) showing the specific labeling of the COOH-terminus of Cx50 (formerly Cx45.6) antibody in many gap junctions in the outer cortical fibers (OC) of the adult chicken lens. The scale bar indicates 500 nm.

**Figure 10:**
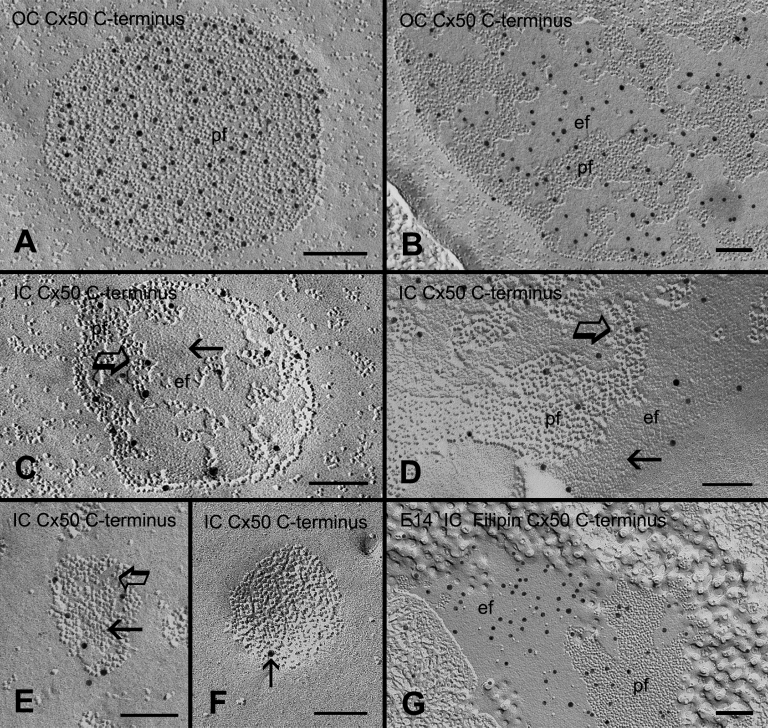
Freeze-fracture immunogold labeling (FRIL) of the Cx50 COOH-terminus in different gap junction configurations. FRIL showing the specific labeling of the COOH-terminus of chick Cx50 antibody in gap junctions in the outer cortex (**A**, **B**) and in the inner cortex (**C**-**F**) at high magnification. In the young outer cortical fibers (OC), immunogold labeling of the Cx50 COOH-terminus antibody can be observed specifically on the P-face (**A**) and E-face (**B**) of gap junctions with loosely-packed connexons. However, a considerably smaller number of immunogold labeling (particle) of the Cx50 COOH-terminus antibody is seen in gap junctions with a mixture of crystalline-packed (arrows) and loosely-arranged (open arrows) connexons in the inner cortex (**C**-**F**). Note that only a single immunogold particle (arrow) is seen in this GJ with crystalline-packed connexons in (**F**). For comparison, in the embryonic chick lens (**G**) in which the gap junctions do not display the distinct crystalline-packed connexons [7], many immunogold particles can be observed in the gap junction with cholesterol-free and non-crystalline-packed connexons. The scale bars indicate 100 nm.

Freeze-fracture immunogold labeling showed that the chick Cx50 antibody generated from the COOH-terminus [46] was labeled in the gap junctions with the loosely-packed connexons in the outer cortical fiber cells (Figure 9 and Figure 10A,B). However, little or no immunogold labeling of the Cx50 COOH-terminus antibody was seen on the gap junctions with tightly- or crystalline-packed connexons in the inner cortex (Figure 10C-F), suggesting that there was a considerable loss of the COOH-terminus of Cx50 in these connexons.

In contrast, the immunogold labeling of Cx50 COOH-terminus antibody was regularly seen in the gap junctions with cholesterol-free and non-crystalline-packed connexons in the embryonic chicken lens (Figure 10G) because the COOH-terminus of Cx50 was preserved in the embryonic chicken lens [36,46,47]. As reported previously [7], these cholesterol-free gap junctions do not display the distinct crystalline-packed configuration of connexons in the embryonic chicken lenses (Figure 10G).

**Figure 11:**
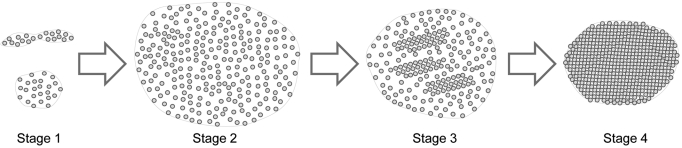
Summary diagram depicting the structural remodeling of gap junctions during fiber cell differentiation and maturation in the adult chicken lens. Stage 1: Formation of small, irregular gap junctions with a few loosely-packed connexons in superficial cortical fiber cells. Stage 2: Growth of a large gap junction with numerous loosely-packed connexons in the young outer cortical fiber cells. Stage 3: Early stage of gap junction remodeling begins in the deeper regions of the outer cortex, displaying several small clusters of crystalline-packed connexons intermingled with a majority of loosely-packed connexons. Stage 4: Mature stage of gap junction remodeling exhibiting distinct hexagonal crystalline-packed connexons observed mainly in the mature inner cortical fiber cells. Due to the compaction of these connexons, these crystalline gap junctions are mostly smaller in size. In concert with these changes, the gap junctions in Stages 1 and 2 are cholesterol-rich, in Stage 3 are cholesterol-intermediate, and in Stage 4 are cholesterol-free. Furthermore, the COOH-terminus of Cx50 is apparently lost in the gap junctions with crystalline-packed connexons in Stage 4.

In summary, Figure 11 depicts the structural remodeling of gap junctions observed during fiber cell differentiation and maturation in the adult chicken lens. The fiber gap junctions apparently undergo several stages of transformation: 1) From small, newly-formed gap junctions (GJs) with a few loosely-packed connexons to the large full-grown GJs with numerous loosely-packed connexons, and both are distributed primarily in the young outer cortical fibers (Stage 1 to Stage 2); 2) The early stage of gap junction remodeling begins in the deeper regions of the outer cortex, displaying several small clusters of crystalline-packed connexons intermingled with a majority of loosely-packed connexons (Stage 3), and 3) the mature stage of gap junction remodeling exhibiting distinct hexagonal crystalline-arranged connexons observed mainly in the mature inner cortical fibers (Stage 4). Due to compaction of these connexons, these crystalline gap junctions are mostly smaller in size. In concert with these changes, the gap junctions in Stages 1 and 2 are cholesterol-rich, in Stage 3 are cholesterol-intermediate, and in Stage 4 are cholesterol-free. Furthermore, the COOH-terminus of Cx50 is apparently lost in the gap junctions with crystalline-packed connexons.

## Discussion

### Changes in the cholesterol redistribution in gap junctions affect connexon packing during fiber cell maturation

By systematic examinations of gap junctions from superficial to deep fiber cells in the adult chicken lens, changes in the connexon packing are seen consistently during the maturation of fiber cells in the adult chicken lens. The gap junctions with the loosely-arranged connexons are mostly distributed in the young outer cortex, whereas those with the tightly- or crystalline-packed connexons are mainly found in the mature inner cortex (Figure 2 and figure 3). Since the changes in connexon packing progress as fiber cells mature, three different configurations of packing can be observed during fiber cell maturation (Figure 2 and Figure 3).The transition between the two distinct structural configurations (i.e., a mixture of the two forms in a given gap junction) can be seen frequently as fiber cells mature, suggesting that these changes are real and they are associated with the fiber cell maturation process of the lens. The similar structural configurations of both mixture and crystalline gap junctions have been reported previously in the adult chicken lens [16]. Furthermore, by using the high pressure freezing procedures in which lens tissues were prepared without using chemical fixation to avoid possible structural artifacts, we have also found the presence of three different connexon packing configurations (Figure 4). In addition, gap junctions with the same crystalline-packed configuration have been regularly found in the deep cortical fibers of the adult rat and mouse lenses using the same high pressure freezing technique in our study (data not shown), suggesting that the structural remodeling of connexons is not species specific.

By using cytochemical detection of cholesterol distribution in gap junctions, the change in connexon packing is shown to correlate well with a gradual loss of cholesterol from gap junctions during fiber cell maturation (Figure 8). It is shown that mobilization (redistribution) of cholesterol within the gap junction can result in creating rows of tightly-packed connexons (Figure 8B,C). The cholesterol molecules, as viewed as filipin cholesterol complexes, can be seen located specifically between the connexon rows. The final removal of these cholesterol molecules from the gap junction would result in the compaction or crystallization of connexon, altering its packing arrangement (Figure 8D,E). Our previous study has shown that the cholesterol molecules are accumulated together in the form of vesicular structures and subsequently removed from gap junctions by endocytosis in the embryonic chicken lens [7]. The similar cholesterol-containing vesicular structures are also regularly found in the fiber gap junctions of adult chicken lenses (data not shown). It is expected that the cholesterol-containing vesicles would be removed by the same endocytotic process from gap junctions in the adult chicken lens.

### Changes in the connexon packing affect connexon density and gap junction area during fiber cell maturation

Due to the significant changes in the connexon packing during fiber cell maturation, the connexon density (i.e., number of connexons per unit area of gap junctions) has been significantly altered (Figure 7). This study shows that the connexon density in the crystalline-packed gap junctions is approximately 2 times higher than that of the loosely-packed ones (Figure 7). This is important because the percentage of membrane area specialized as gap junctions in the outer cortex is approximately 5 times greater than that of the inner cortex (Figure 6). This result suggests that the large decrease in the membrane area specialized as gap junctions from the outer cortex to inner cortex would be compensated considerably by the significant increase of the connexon density in the inner cortex during fiber cell maturation. This means that although the total area of gap junctions decreases as fiber cells mature, the number of connexons and their communications would not be decreased by the same magnitude in the mature inner fibers.

### Possible functional significance for the structural remodeling of gap junctions during fiber cell maturation

Due to dramatic changes in the membrane surface structures from the outer to inner cortex during lens maturation and aging, the distribution of smooth membrane surface is significantly reduced in the inner cortical fiber cells [48-56]. The narrow undulating and bumpy membrane surfaces in the inner cortex would not be favorable for distribution of large gap junctions frequently seen in the outer cortical fiber cells (Figure 2 and Figure 5). This is evidenced by the distribution of the single large-size group of gap junctions (i.e., 0.8-1.75 μm^2^) observed only in the outer cortex but not in the inner cortex (Figure 5). It is this group of the large-size gap junctions that contributes largely to the significant difference (i.e., ~5 times difference) in the percentage of membrane area specialized as gap junctions in the outer cortex as compared with the inner cortex (Figure 6). However, the large decrease in gap junction area is compensated considerably by the increase (i.e., ~2 times increase) in the connexon density of gap junctions in the inner cortex during fiber cell maturation (Figure 7). This compensation is done by the change in the connexon packing of gap junctions from the outer to inner cortex during fiber cell maturation as presented in this study. It is clear that the gap junction remodeling plays an important role in the increase in the connexon density to ensure adequate cell-to-cell communications required for the normal function of mature cortical fiber cells in the adult chicken lens.

In addition, our freeze-fracture immunogold labeling study indicates that the transformation of loosely- to crystalline-packed connexons is also associated with the loss (cleavage) of the COOH-terminus of Cx50 during fiber cell maturation (Figure 10). It is conceivable that the loss of the Cx50 COOH-terminus (163 amino acids) of 18.2 kDa molecular weight would facilitate the physical intimacy between adjacent connexon molecules in the cholesterol-free gap junctions. A large-scale loss of the Cx50 COOH-terminus in the cholesterol-free gap junctions may ultimately result in the formation of the tightly- and crystalline-packed configurations of connexons. This possibility would explain why the cholesterol-free gap junctions in the embryonic chicken lens do not display the same distinct crystalline-packed connexons because the COOH-terminus of Cx50 is not cleaved in the inner cortex of the embryonic chicken lens [36,46,47,57].

The loss of the COOH-terminus of Cx50 has been implicated for the unique physiological change in response to the different pH environments during fiber cell maturation in the lens. It is known that gap junctions in the superficial differentiating fiber zone are pH sensitive, while those in the mature inner fiber zone are pH insensitive [9,35,37,58-62]. It has been postulated that because, deep in the lens, the fiber cell cytoplasm is significantly more acidic (pH 6.81), the loss of gap junction sensitivity to pH may be crucial to keeping the gap junction channel open for lens survival [58,59]. Several studies have suggested that the loss of the COOH-terminus of Cx50 is responsible for its pH sensitivity change from the young superficial fibers to mature deeper fibers in adult lenses [32,35-37].

In conclusion, this study has demonstrated that the structural remodeling of gap junctions during fiber cell maturation in the adult chicken lens differs significantly from the one seen in the embryonic lens. The distinct change in the connexon packing from the loosely- to crystalline-packed configurations can result in the increase of connexon density in the mature cortical fiber cells. It appears that the formation of crystalline-packed connexons is facilitated by the removal of cholesterol and the loss of the Cx50 COOH-terminus from gap junctions in the adult chicken lens.
